# Identification of novel proteins involved in P2X7-mediated signaling cascades

**DOI:** 10.1007/s11302-022-09893-z

**Published:** 2022-08-12

**Authors:** Lukas Sassenbach

**Affiliations:** Walther-Straub-Institute of Pharmacology and Toxicology, Faculty of Medicine, LMU Munich, Munich, Germany

**Keywords:** P2X7, Phosphatidylserine, EROS, XKR scramblase, Vsp13a

## Abstract

High concentration of extracellular ATP acts as a danger signal that is sensed by the P2X7 receptor (P2X7R). This ATP-gated ion channel has been shown to induce multiple metabotropic events such as changes in plasma membrane composition and morphology, ectodomain shedding, activation of lipases, kinases, and transcription factors as well as cytokine release. The specific signaling pathways and molecular mechanisms remain largely obscure. Using an unbiased genome-scale CRISPR/Cas9 screening approach in a murine T cell line, Ryoden et al. (2022, 2020) identified three proteins involved in P2X7 regulation and signaling: Essential for Reactive Oxygen Species (EROS) is essential for P2X7 folding and maturation, and Xk and Vsp13a are required for P2X7-mediated phosphatidyl serine exposure and cell lysis. They further provide evidence for an interaction of Xk and Vsp13a at the plasma membrane and confirm the role of Xk in ATP-induced cytolysis in primary CD25^+^CD4^+^ T cells from Xk^−/−^ mice.

## Commentary

P2X receptors (P2XRs) represent a family of trimeric, non-selective ion channels. The homomeric P2X7 subtype differs structurally and functionally from other P2X family members. Its ATP sensitivity is 10- to 100-fold lower and is therefore considered to act as a “danger signal” detector at sites of damaged tissue [1]. P2X7 activation initiates diverse signaling cascades resulting in cytokine release, plasma membrane reorganization, ectodomain shedding, and cell death. The molecular mechanisms are poorly understood but have been associated with its large intracellular C-terminus that is absent in other P2XRs [2]. Multiple interaction motives and protein interactions of the tail have been proposed but few have been confirmed and the molecular mechanisms of downstream signaling remain unknown. A recent breakthrough was the determination of the cryo-EM structure of the full-length rat P2X7R, which revealed a densely packed intracellular globular “ballast domain” in which many of the proposed interaction sites are hidden [3]. Unexpectedly, this domain contains a GTP binding motive as well as a dinucelar Zn^2+^ binding site, suggesting a metabotropic function. In a recent paper, the Nagata group investigated the mechanism of ATP-mediated phosphatidylserine (PtdSer) exposure in T lymphocytes. To this aim, the group created a TMEM16F^−/−^ P2X7^−/−^ double knockout (DKO) T cell lymphoma (WR19L) cell line, which they transformed with the murine P2X7k variant, the predominant splice form of P2X7 in WR19L WT cells [4] which is also more sensitive to ATP [5]. TMEM16F, also called Anoctamin6 (Ano6), is part of the transmembrane protein 16 (TMEM16) family and functions as both a Ca^2+^-dependent phospholipid scramblase and Ca^2+^-activated Cl^−^ channel [6, 7]. It has previously been shown to mediate several P2X7-activated and Ca^2+^-mediated responses in macrophages and HEK293T cells [8, 9] but was not essential in the T cell line. The DKO-mP2X7k-cells were then used for an adapted genome-scale CRISPR-Cas9 knockout screen initially described by Salem et al. [10]. Shortly, this approach uses a genome-scale CRISPR knockout (GeCKO) library that contains 130,209 single guide RNA (sgRNAs) against 20,611 mouse genes [11]. Cells transfected with the GeCKO library were stimulated with the P2X7-agonist BzATP and sorted for absence of PtdSer exposure determined by Annexin V staining. Annexin V–negative cells were subsequently analyzed via deep sequencing of their chromosomal DNA to identify the enriched incorporated sgRNA. Next-generation sequencing (NGS) identified 34 genes for which four to six unique sgRNA sequences were present. Most of them were targeted against EROS, while sequences against Xk and Vsp13a ranked second and third (after exclusion of supposed artifacts) [12].

EROS was first described in 2017 by Thomas et al. and was shown to be essential for host defense via the phagocyte nicotinamide adenine dinucleotide phosphate oxidase (NADPH oxidase) by affecting the abundance of the membrane bound heterodimer of the multi-subunit complex NADPH oxidase [13]. It remained unclear whether EROS might also have a regulatory effect on the expression of other proteins. Its involvement in P2X7 regulation was recently independently confirmed by Randzavola et al. [14], using a tandem Mass Tag proteomics approach of macrophages and CD4^+^ T lymphocytes from both WT and EROS^−/−^ mice. In these unbiased screens, it was found that EROS^−/−^ cells showed reduced P2X7 levels and therefore smaller P2X7-mediated cellular response toward ATP stimulation. This includes impaired phospholipid scrambling and dye-uptake in (DKO)-WR19L cells, reduced IL-1ß production of LPS primed human THP-1 derived macrophages and murine bone marrow–derived macrophages (BMDM), reduced caspase-1 activation in macrophages, and impaired CD62L shedding in CD4^+^ T cells [4, 14]. On the other hand, overexpression of EROS increased P2X7 abundance in the plasma membrane. Both Randzavola et al. [14] and Ryoden et al. [4] also presented evidence for a direct interaction between EROS and P2X7 by co-immunoprecipitation of P2X7 with EROS in RAW264.7 macrophages and WR19L cells, a structural complementation reporter system and confocal microscopy. While they found P2X7 mainly localized in the plasma membrane, an overlapping signal with EROS was identified in the ER. Together, these papers show convincing evidence that EROS is of importance for P2X7-mediated cellular response and most likely acts as a chaperone, assisting P2X7 folding and maturation in the ER before it can be transported to the plasma membrane.

The second and third most abundant sgRNAs in the screen from Ryoden et al. are targeted against Xk and Vps13a. Mutations of these proteins are linked to two neurodegenerative syndromes with similar phenotypes, the McLeod syndrome (MLS) and chorea acanthocytosis (ChAc), respectively [15, 16]. Similar to EROS, knockout of Xk or Vps13a in DKO-mp2X7k-WR19L cells reduces BzATP-induced PtdSer exposure. Contrary to EROS though, their respective knockouts do not affect the expression or total abundance of P2X7 on the plasma membrane, implying that both proteins are important for the BzATP-induced signaling cascade that leads to PtdSer exposure. Xk has been shown to be part of the Xkr family of caspase-dependent scramblases [17]. Vps13a is a member of the VPS13 family. Members of this transmembrane protein family have been shown to form conduits between membranes of different organelles, allowing lipid transfer [18]. Ryoden et al. [12] used BN-PAGE, western blotting, and confocal microscopy to show that Xk and VPS13a form a complex within the plasma membrane and that the majority of Vps13a is associated with Xk. Additional studies support this interaction: Erythrocytes of McLeod syndrome showed that lack of Xk leads to a loss of Vsp13a in their membrane [19] and overexpression of Xk has been shown to relocalize overexpressed Vps13a from contacts between lipid droplets and the ER to the entirety of the ER together with Xk in HEK293T cells [20]. Recently this interaction has been further characterized: Using AlphaFold, two independent studies show that the pleckstrin homology (PH) domain of Vps13a and the second intracellular loop of Xk mediate the suggested interaction [27, 28]. Finally, Ryoden et al. were able to show that absence of Xk delays the PtdSer exposure as well as necrotic cell death of CD25^+^CD4^+^ T cell isolated from Xk^−/−^ mouse spleens by 40% compared to WT cells [12].

The asymmetric distribution of PtdSer and other phospholipids between inner and outer leaflet of the plasma membrane bilayer is maintained by P4-type ATPases actively positioning them to the inner leaflet [21–23]. During apoptosis or platelet aggregation, these ATPases are inactivated either directly by intracellular Ca^2+^ increase or indirectly by caspase activation and cleavage of intracellular domains [24]. These same stimuli simultaneously activate the phospholipid scramblases Xkr8 or TMEM16 that actively yet unspecifically translocate phospholipids in both directions. This then leads to an increased exposure of PtdSer to the plasma membrane surface where it acts as an indicator for apoptosis or platelet aggregation [25, 26]. TMEM16F has previously been reported to influence P2X7 downstream effects such as PtdSer exposure in HEK293T cells and membrane blebbing and cell shrinkage in macrophages [8]. A functional interaction between P2X7 and TMEM16F was recently supported by studies in HEK293T cells and *Xenopus* oocytes where an influence on macropore formation and current facilitation, both hallmarks of P2X7 activation, was found [9]. An intriguing question is whether the different findings of Ryoden et al. [12] and Ousingsawat et al. [8] can be explained by the use of different cell types. Also, the specific molecular mechanisms and the question if direct protein interactions occur requires further investigation.

In summary, using an adapted genome-scale CRISPR/Cas9 Screening protocol, Ryoden et al. identified three proteins, EROS, Xk, and Vps13a, that are important for P2X7-mediated PtdSer exposure. They show that EROS acts as a chaperone for P2X7, regulating the abundance of P2X7 in the plasma membrane, while Xk and Vps13a seem to be involved in downstream signaling of P2X7. Although they were able to show that Xk and Vps13a form a complex, the question how they are activated in the P2X7-mediated signaling cascades remains elusive as the increasing intracellular levels of Ca^2+^ mediated by P2X7 are not essential for PtdSer exposure [4]. Whether the Xk-Vps13a-complex is activated through another downstream event of P2X7 activation or whether they are interacting with P2X7 directly remains unknown.

## Data Availability

Not applicable.
